# Be Up and Doing

**Published:** 1897-11

**Authors:** 


### EDITORIAL.

### BE UP AND DOING.

THIS will be a winter's season of important meetings all over the country, and we wish to urge upon every veterinarian to attend them as far as possible. From the meeting of the American Public Health Association in Philadelphia; an important meeting of the Massachusetts Veterinary Association, to be addressed by Prof. Theobald Smith on "The Restriction of Tuberculosis among Dairy-cattle" in October; in November, a special meeting of the Woman's Health Protective Association of Philadelphia, and kindred organizations to take up the subject of better meat-inspection and the abolition of private slaughter-houses. Equally important meetings in New York City and Washington on similar subjects are in contemplation, all of which will be fruitful of good for the veterinary profession in a just proportion corelative with the interest they take in them. Let every one keep in touch with these movements, and the winter's work will be profitable for our profession.

